# The economic costs of a multisectoral nutrition programme implemented through a credit platform in Bangladesh

**DOI:** 10.1111/mcn.13441

**Published:** 2022-10-18

**Authors:** Giang Thai, Amy Margolies, Aulo Gelli, Nasrin Sultana, Esther Choo, Neha Kumar, Carol Levin

**Affiliations:** ^1^ International Food Policy Research Institute Washington District of Columbia USA; ^2^ USA International Food Policy Research Institute Washington District of Columbia; ^3^ Independent Consultant Dhaka Bangladesh; ^4^ Department of Global Health University of Washington Seattle Washington USA

**Keywords:** costs, international child health nutrition, low income countries, maternal public health, nutritional interventions, programme evaluation

## Abstract

Bangladesh struggles with undernutrition in women and young children. Nutrition‐sensitive agriculture programmes can help address rural undernutrition. However, questions remain on the costs of multisectoral programmes. This study estimates the economic costs of the Targeting and Re‐aligning Agriculture to Improve Nutrition (TRAIN) programme, which integrated nutrition behaviour change and agricultural extension with a credit platform to support women's income generation. We used the Strengthening Economic Evaluation for Multisectoral Strategies for Nutrition (SEEMS‐Nutrition) approach. The approach aligns costs with a multisectoral nutrition typology, identifying inputs and costs along programme impact pathways. We measure and allocate costs for activities and inputs, combining expenditures and micro‐costing. Quantitative and qualitative data were collected retrospectively from implementers and beneficiaries. Expenditure data and economic costs were combined to calculate incremental economic costs. The intervention was designed around a randomised control trial. Incremental costs are presented by treatment arm. The total incremental cost was $795,040.34 for a 3.5‐year period. The annual incremental costs per household were US$65.37 (Arm 2), USD$114.15 (Arm 3) and $157.11 (Arm 4). Total costs were led by nutrition counselling (37%), agriculture extension (12%), supervision (12%), training (12%), monitoring and evaluation (9%) and community events (5%). Total input costs were led by personnel (68%), travel (12%) and supplies (7%). This study presents the total incremental costs of an agriculture‐nutrition intervention implemented through a microcredit platform. Costs per household compare favourably with similar interventions. Our results illustrate the value of a standardised costing approach for comparison with other multisectoral nutrition interventions.

## Key messages

- Nutrition‐sensitive agriculture programmes can improve rural undernutrition but lack information on costs.
- We use a standardised approach to estimate the total incremental costs of an integrated nutrition intervention in Bangladesh to improve maternal and child undernutrition.
- Costs per household compare favourably with similar interventions.
- This study provides evidence on the costs of integration to support the design and implementation of multisectoral nutrition programmes.
- Our results illustrate the value of standardising costing to facilitate comparisons with other multisectoral nutrition interventions.

## Graphical abstract

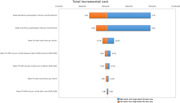

## 1 BACKGROUND

Child and maternal malnutrition is a persistent problem in Bangladesh. The prevalence of stunting in Bangladesh is 31% for children under 5, with 9% severely stunted and 2% severely wasted. Further, children in rural areas of the country are more likely to be stunted than their counterparts in urban areas. In Bangladesh, 24% of ever‐married women between the ages of 15−19 years of age are undernourished and rural women are more likely to suffer from undernourishment than urban women. At the same time, the proportion of overweight women has increased to 32%, highlighting the problem of both under and overnutrition in the country (NIPORT & ICF, 2020).

Nutrition‐specific interventions that address the immediate causes of malnutrition have long been used to target undernutrition. Nutrition‐sensitive interventions—that address intermediate or underlying causes of malnutrition and address multiple outcomes—have also shown promise. Nutrition‐sensitive agriculture (NSA) programmes, in particular, have the potential to accelerate progress in addressing child malnutrition (Ruel & Alderman, 2013). Effective NSA programmes often combine behaviour change communication (BCC) with the production of nutrient‐rich foods, increasing dietary diversity and improving caregiver knowledge and infant and young child feeding practices in poor households (Keats et al., 2021). Engaging women in agriculture and nutrition can increase their decision‐making power and control over assets, albeit with potential tradeoffs in work burdens. Women's empowerment in Bangladesh has also been linked to improvements in diet diversity and reductions in child stunting (Holland & Rammohan, 2019). Growing evidence shows that well‐implemented nutrition‐sensitive agricultural programmes can improve maternal and child diets and household access to nutritious foods (Ruel et al., 2018). However, a key challenge for policymakers and programme implementers prioritising investment decisions hinges on the gap in the evidence on the costs of implementing multisectoral agriculture‐nutrition programmes (Ruel & Alderman, 2013; Ruel et al., 2018). This is further limited by a lack of standardised methods that make comparisons difficult to interpret (Gyles et al., 2012; Njuguna et al., 2020; Ramponi et al., 2021).

The Targeting and Realigning Agriculture to Improve Nutrition (TRAIN) project was a multisectoral nutrition‐sensitive intervention implemented in rural Bangladesh by the nonprofit Building Resources Across Communities (BRAC). The intervention was designed to be evaluated by a randomised controlled trial (RCT). The RCT baseline survey was implemented in 2016−2017 by the International Food Policy Research Institute (IFPRI).

This study presents the incremental costs of implementing an integrated agriculture‐nutrition intervention through an existing BRAC microcredit platform.^1^ We estimate the total incremental financial and economic costs of the TRAIN programme by implementation arm, including costs per beneficiary. We also examine cost shares by programme inputs and activities.

Financial costs represent the implementing partner's actual expenditures on goods and services purchased to deploy the intervention. Economic costs, on the other hand, are defined as the opportunity cost of all of the resources used to produce something; and can include the value of resources that may not have been paid for, such as volunteer frontline worker time or programme participant time. Once data becomes available from an ongoing impact evaluation, these costs will be combined with programme benefits for a full economic evaluation including cost‐benefit and cost‐effectiveness analyses.

Our findings serve many purposes. A robust understanding of the costs of multisectoral nutrition strategies is critical for priority‐setting and for motivating donors. This study is useful for governments and development partners to target investments in multisectoral nutrition programmes. Standardised unit cost data provides a cost benchmark for governments, donors and non‐profit organisations on how to design, budget and measure the resource requirements of interventions. Lastly, this analysis contributes to an effort to build an evidence base on the costs of multisectoral nutrition programmes across different settings and platforms. This study was conducted by the Strengthening Economic Evaluation for Multisectoral Strategies for Nutrition (SEEMS‐Nutrition) consortium, led by the University of Washington in partnership with IFPRI. SEEMS‐Nutrition develops standardised approaches and tools to assess the costs and benefits of multisectoral nutrition programmes.

### 1.1 The TRAIN intervention

TRAIN was a 3.5‐year project (2016−2020) designed to address evidence gaps on agriculture‐based interventions to improve maternal and child nutrition. BRAC started the programme in 2016 in the Dhaka, Khulna and Rangpur divisions. A total of 5040 households were selected from 144 unions of 36 Subdistricts from 10 districts. TRAIN incorporated a BCC strategy for maternal and child health and nutrition into a female‐focused microcredit programme promoting production diversity and income generation.

BRAC has led microcredit programmes in Bangladesh since 1974. Dabi, a credit platform lending only to women, has coverage through 2146 local branches with more than 3.5 million borrowers. Dabi provides loans to women to increase income and production in agriculture and to promote empowerment. Dabi disburses $1.8 billion in loans annually, the majority for agriculture (60%).^2^ The TRAIN programme was built upon the existing Dabi microcredit platform linked to agriculture. Programme criteria dictate that one member of the household is a married female Dabi beneficiary (henceforth referred to as the ‘index female’) of childbearing age between 15 and 49 years old. If there was more than one woman with qualifying criteria in the household, then one women was selected randomly.

The TRAIN programme had an additive design with three different treatment arms and a control group. Each arm built an additional component onto the control arm (the existing Dabi microfinance platform) (Supporting Information: Appendix Figure 1). Arm 2 incorporated nutrition BCC to address constraints to caregiver nutrition knowledge and practices. In arm 3, household visits with agricultural extension and training were added to arm 2 components. Arm 4 incorporated all other components plus gender sensitisation to increase awareness of women's health and nutrition and their productive and reproductive roles. Gender activities included community‐level gender forums and household‐level gender counselling. Two cadres of frontline workers conducted programme activities and delivered BCC messaging to beneficiary households. Nutrition frontline workers (Pushti Kormis—PKs) provided messages on women's nutrition, infant and young child feeding and water, sanitation and hygiene through home visits and community events. Field Organisers (FOs) delivered agricultural extension to households and led gender activities at the community level. FOs also supervised PKs. Other personnel included BRAC District Managers (DMs) who managed the programme and supervised frontline workers.

TRAIN had a 6‐month start‐up period (October 2016−March 2017). This paper presents the cost analysis from 40 months (3.5 years) of programme implementation (October 2016−January 2020). Given that the last 3 months of implementation were disrupted by COVID‐19 and extended until October 2020, our study only covers the pre‐COVID period.

## 2 METHODS

This study used a novel standardised costing approach that contributes to gaps in the literature on the costs of multisectoral nutrition programmes (Margolies et al., 2021). The methodology was developed by SEEMS‐Nutrition, led by the University of Washington in collaboration with IFPRI and funded by the Bill and Melinda Gates Foundation. The SEEMS‐Nutrition approach provides standardised research protocols, data collection tools and guidance on allocating costs. This approach defines a set of input and activity cost category codes that are specific to multisectoral intervention components for agriculture, nutrition and gender empowerment (C. Levin et al. 2019). Our analysis also adheres to principles outlined in the Global Health Cost Consortium Reference Case for Estimating the Costs of Global Health Services and Interventions (Vassall et al., 2017).

The cost analysis was conducted from the payer and societal perspectives. The analysis included costs incurred by BRAC, frontline workers and programme beneficiaries. Costs related to third‐party external research were excluded. The SEEMS‐Nutrition framework uses a four‐step approach. Costs are aligned with a multisectoral nutrition typology that identifies resource use and outputs along the programme impact pathways to achieve standardised unit costs and the basis for benchmarking and economic evaluation. Step 1 aligns the TRAIN programme to a typology of nutrition‐sensitive value (NSV) chain interventions that (1) increase the supply of nutrient‐rich foods, (2) increase the demand for nutrient‐rich foods, and (3) promote the enabling environment for nutrition. Step 2 maps the programme impact pathways to clearly articulate the linkages from activities to outputs and outcomes. Step 3 identifies all activities, inputs and costs along the impact pathway. Step 4 identifies outputs and outcomes for each activity to define the components of total and unit costs. The four‐step framework estimates the direct intervention costs and opportunity costs associated with all programme activities.

Multisectoral nutrition approaches may include one or more of the intervention typologies with different components, services and outputs. Therefore, it is important to clearly define the unit cost for one or more outputs. Supporting Information: Appendix Table 1 shows the standardised process to derive the unit cost per beneficiary. Supporting Information: Appendix Tables 2 and 3 describe the standardised SEEMS‐Nutrition activity and input categories and definitions. Supporting Information: Appendix Table 4 illustrates how costs were mapped to the NSV chain typology. NSV chain interventions are designed to increase nutritional value along the smallholder supply chain. The NSV typology provides a framework for comparing intervention strategies and entry points (De la Pena et al., 2020). The use of the typology also facilitates comparisons with other interventions costed with the SEEMS‐Nutrition framework. Further details of cost calculations (Supporting Information: Tables 5−7) and data collection are found in the Appendix (Supporting Information on data collection).

### 2.1 Data collection

The cost analysis captures the total costs of the TRAIN intervention incremental to the existing microcredit programme. Primary and secondary cost data were collected for the period of October 2016−January 2020, which included 6 months of start‐up and 3 years of full implementation. The SEEMS mixed‐methods approach combines financial expenditure data with micro‐costing methods to identify and value resources and allocate costs to activities and inputs. The Activity‐Based‐Costing‐Ingredients (ABC‐I) method (Kaplan & Anderson, 2004; Tan‐Torres Edejer et al., 2003) for micro‐costing has been previously applied to nutrition programmes to assess cost‐efficiency and cost‐effectiveness (Fiedler et al., 2008; Heckert et al., 2019; C. Levin et al. 2019; Margolies & Hoddinott, 2015). Costs were obtained from existing records, surveys and from primary data collection with BRAC.

We leveraged secondary data from programme monitoring and reports. When available, planning and progress reports were reviewed retrospectively for randomly selected programme staff and frontline workers. Costs were disaggregated into start‐up and recurrent categories. Start‐up costs occurred in the first 6 months, such as planning, materials development and staff training. Recurrent costs included ongoing activities like household visits, community events and monitoring.

Primary cost data were collected in two rounds (May 2019 and February 2020) through semistructured in‐depth interviews (IDIs) and focus group discussions (FGDs). IDIs and FGDs gathered data on interviewees' opportunity costs and out‐of‐pocket (OPP) expenses. In the first round of data collection, we organised IDIs and FGDs at a centralised location. FGDs were conducted with FOs (*n* = 1) and DMs (*n* = 1), and IDIs with BRAC staff (*n* = 3) and PKs (*n* = 3). In the second round, 7 additional FGDs were conducted at BRAC regional offices in Rangpur (*n* = 4), Dhaka (*n* = 2), and Khulna (*n* = 4) divisions with PKs for a total of 24 participants. One FGD was conducted with FOs (*n* = 2) and one FGD was conducted with DMs (*n* = 5). Additional IDIs were conducted with BRAC head office staff (*n* = 4). A local research collaborator facilitated and translated interviews in the local language. Supporting Information: Appendix Table 7 provides further primary data collection details.

We estimated the opportunity cost of beneficiary participation with data from the process evaluation. The process evaluation included information on beneficiary time use for the index female respondent and the index husband in each household.^3^ The process evaluation was conducted by IFPRI in April 2019. Programme output data were collected from the RCT baseline survey and from BRAC monitoring surveys. These included the number of beneficiaries reached by each activity. These data provided the denominator for the unit cost calculations. Table 2 provides a summary of unit costs including beneficiary and output data.

Data were stored in secure password‐protected computers and servers according to IFPRI and BRAC data governance policies. The data were only accessible to the research team. All hard copies of consent forms, structured interviews and timesheets were kept in a safe location only accessible to the research team. Ethical clearance for the impact evaluation including cost analyses was obtained from the IFPRI Institutional Research Board, BRAC University, Bangladesh, and the Bangladesh Medical Research Council. The study was registered on the Registry for International Development Impact Evaluation.

### 2.2 Data analysis

We analysed secondary expenditure and process evaluation data and combined these with primary data on economic costs. First, we analysed process evaluation data on beneficiary time allocation and OOP expenditures for participating households using Stata 16 statistical software. Second, we obtained financial expenditure data from BRAC. These data were entered into a SEEMS‐Nutrition expenditure analysis template in Microsoft Excel (Version 16). We then mapped line‐item expenditures to standardised input and activity codes using the template. Third, we used Excel to summarise and analyse micro‐costing data from the qualitative interviews and focus group discussion.

Most line‐item expenditures were easily mapped to standardised SEEMS activity and input codes. However, there were some exceptions: (1) BRAC personnel who contributed to multiple activities; and (2) shared capital and supply costs. For the first, we developed allocation rules for each activity using data from KIIs and FGDs. For example, using qualitative interviews we found PKs spent 80% of their time on nutrition counselling, 10% on training, 5% on planning and 5% on coordination meetings. PK salaries were allocated accordingly to those activities. Shared inputs or capital costs were allocated proportionally across the related activities. Shared costs are described in greater detail below.

#### 2.2.1 Personnel costs

First, all nonshared financial and economic personnel costs were allocated across programme activities. We combined expenditure and time allocation data from BRAC staff and frontline workers (IDIs and FGDs). This information was used to allocate personnel costs to programme activities (Supporting Information: Appendix Table 2). Since the intervention was built upon ongoing activities, only one national staff—a Senior Sector Specialist ‐ was assigned to the programme full‐time. We interviewed this person to obtain information on their time allocation to programme activities. At the subnational level, BRAC staff were assigned part‐time. Therefore, we first captured the share of subnational staff time spent supporting TRAIN versus other BRAC programmes. For example, for each DM we estimated the total minutes per year spent on TRAIN to calculate a percentage of annual time spent for our estimations. On average, DMs spent 30% full‐time equivalent on TRAIN activities annually. Next, we used estimates of annual time dedicated to TRAIN and allocated time to individual activities such as home visits. Personnel costs also included additional travel time and OOP costs for frontline workers (PKs) beyond the 36‐h week paid by BRAC.

#### 2.2.2 Economic costs of frontline workers

Estimates of frontline worker (PK) costs include personnel costs from BRAC expenditures combined with estimates of OOP costs and the valuation of time above the contracted 36‐h week. Economic costs such as OOPs and overtime hours/travel were not reimbursed by BRAC. PK personnel costs, gleaned from both financial expenditure and economic cost data were allocated based on time spent on programme activities.

#### 2.2.3 Beneficiary opportunity costs

Beneficiaries participated in programme activities such as household counselling and community events. We estimated the opportunity costs of participation in TRAIN activities from process evaluation data. Opportunity costs were based on information on beneficiaries’ OOP expenses and the average time per year spent on programme activities. To value beneficiaries' time accurately, we used daily wage rates for agricultural labour for men and women from a 2017 IFPRI survey in implementation villages. We used mean daily wage rates for male and female agricultural workers to value beneficiaries' time. Once we obtained all personnel and beneficiary costs aligned to programme activities, we mapped them onto the standardised SEEMS activity categories (Supporting Information: Appendix Table 2).

#### 2.2.4 Start‐up and capital costs

One‐time start‐up costs, capital and equipment costs for durable goods valued over USD$100 and lasting over 1 year were annuitized. These costs were annuitized over the implementation period using a discount rate of 3% and an expected useful life of 10 years. Annuitization ensures an equivalent annual cost is estimated and reflects the value‐in‐use of capital items, rather than reflecting the financial cost from the time of purchase (Brooker et al., 2008). Taxes for durable goods and value‐added taxes for small goods where tax was included as part of the commodity cost were included in the financial costs. Taxes were excluded in economic costs except in the case of small supplies. Costs were adjusted for inflation and are presented in 2019 USD using an exchange rate of $1USD/84.77 Bangladesh taka (BDT).^4^

#### 2.2.5 Unit costs

Total incremental costs were broken down by their financial and economic components. The total incremental cost per beneficiary is defined as the total cost divided by the total number of beneficiaries. We also present cost breakdowns by intervention typology, programme activity, inputs and timing (start‐up and recurrent). We estimate annual cost per beneficiary and the annual cost per household by treatment arm. The cost profile is the share of the disaggregated cost over the total programme costs for the 3.5‐year period.

#### 2.2.6 Sensitivity analyses

A Monte Carlo probabilistic sensitivity analysis was performed using Oracle's Crystal Ball software (https://www.oracle.com/applications/crystalball). This technique produces estimates of thousands of possible scenarios, generating ranges of potential outcomes and identifying inputs that are impactful on the model. Distributions were assigned to the following parameters: (i) variation in the amount of frontline worker (Pushti Kormis, or PK) overtime, (ii) variation in frontline worker (PK) OOP costs and (iii) variation in beneficiary opportunity costs (Table 3). We used a gamma distribution for all parameters since hours worked and costs are all nonnegative (Dodd et al, 2006). For each scenario, 5,000 simulations were run with samples drawn from the various parameter distributions. We provide the below rationale and details for each parameter we varied in the sensitivity analysis. See Supporting Information: Appendix Table 6 for additional details.

1. Overtime and OOP expenses for frontline workers (PKs): The estimate of overtime includes labour and travel time that was not reimbursed during the salaried work week. PK overtime varied by geographical location and by which treatment arm they supported with their activities. Variation in overtime by treatment arm was due to the additive design of the intervention. For example, Arm 4 involves more programme activities than Arm 2 or 3. The geographical spread of intervention households and the frontline workers' ability to afford transportation costs also affected their time investments. Variations in frontline worker overtime and OOP expenses were simulated using the means and standard deviations from actual data collected from the sample of PKs converted into shape and scale parameters for the gamma distribution.
2. Beneficiary opportunity costs: Beneficiary participation in the programme was estimated using process evaluation data on the amount of time spent on TRAIN activities. The baseline survey provided local wage rates for men and women. The sensitivity analysis varied opportunity costs separately for men and women using means and standard deviations from the process evaluation and assumes a gamma distribution (Table 3).

## 3 RESULTS

The total incremental cost of the TRAIN programme including economic and financial costs over 3.5 years was USD$795,040.34 (Table 1). The financial costs were USD$598,578, accounting for 75.3% of total costs. Economic costs totalled USD$196,461 or the remaining 24.7% of total costs. Start‐up costs were USD$37,950.02, or 5% of total costs, while the remaining 95% of total costs were recurrent (USD $757,090.32). The total incremental cost per household for the programme duration ‐ regardless of treatment assignment—was USD$210. The average cost per household, regardless of treatment assignment, was USD$63.10. The average cost per beneficiary, which included the index female of the household and their spouse, regardless of treatment assignment, was USD$33.64.

**Table 1:** Summary of total incremental costs for the TRAIN intervention (USD, 2019)

	Financial cost (USD)	Economic cost (USD)	Total cost (USD)	%
Input				
Personnel (hired)	$362,654.50	$19,014.29	$381,668.79	48%
Personnel (beneficiaries)		$162,335.76	$162,335.76	20%
Supplies	$56,250.25		$56,250.25	7%
Equipment (capital goods, including vehicles)				0%
Agriculture supplies	$2196.27		$2196.27	0%
Agriculture equipment	$5.81		$5.81	0%
Contracted services	$49,150.36		$49,150.36	6%
Fuel and maintenance	$19.69		$19.69	0%
Travel/per diem/allowances	$81,937.52	$15,111.39	$97,048.91	12%
Overhead	$46,364.50		$46,364.50	6%
Total	$598,578.91	$196,461.43	$795,040.34	100%
Stage				
Start‐up	$37,950.02		$37,950.02	5%
Recurrent	$560,628.89	$196,461.43	$757,090.32	95%
Total	$598,578.91	$196,461.43	$795,040.34	100%
Activity				
Planning/microplanning	$32,678.56	$757.08	$33,435.64	4%
Programme Installation	$1171.23		$1171.23	0%
Awareness raising/sensitisation	$1089.92		$1089.92	0%
Training	$74,981.41	$17,293.66	$92,275.07	12%
Materials development	$10,833.70		$10,833.70	1%
Management	$4,493.32		$4493.32	1%
Monitoring and evaluation	$72,568.92	$2,649.77	$75,218.70	9%
Procurement	$6724.76		$6724.76	1%
Distribution of inputs	$5050.17		$5050.17	1%
Site supervision	$93,101.67		$93,101.67	12%
Home visits: nutrition/gender	$161,760.07	$128,936.16	$290,696.23	37%
Home visits: agriculture/poultry extension	$60,323.57	$33,049.53	$93,373.10	12%
Community events/extension	$30,139.95	$13,447.68	$43,587.63	5%
Integration and coordination	$11,076.85	$327.56	$11,404.41	1%
Indirect/overhead	$32,584.80		$32,584.80	4%
Total	$598,578.91	$196,461.43	$795,040.34	100%

Abbreviation: TRAIN, Targeting and Realigning Agriculture to Improve Nutrition.

### 3.1 Cost drivers

**Figure 1:**
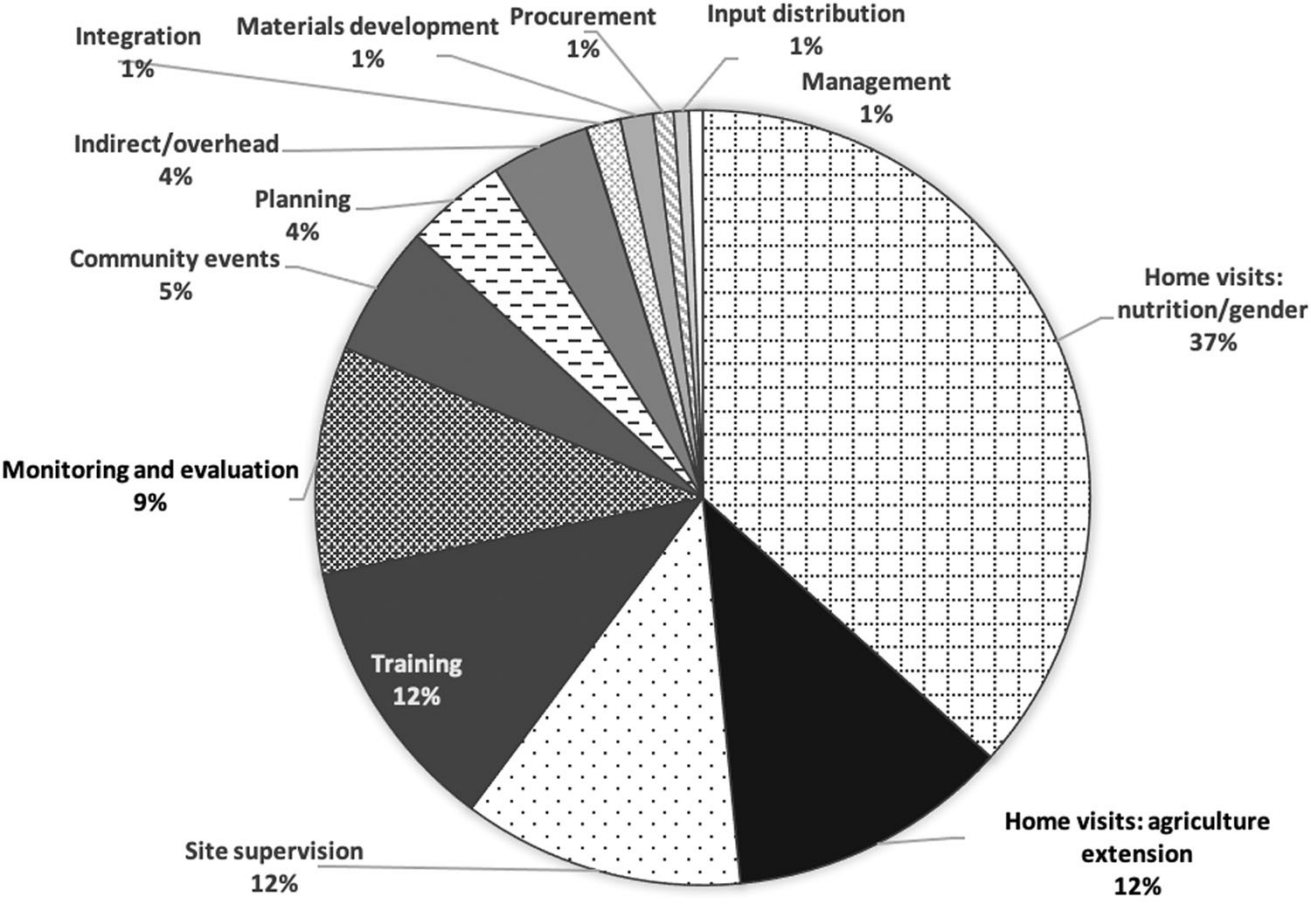
Cost drivers for the TRAIN intervention in Bangladesh by activity type (Source: Authors). TRAIN, Targeting and Realigning Agriculture to Improve Nutrition.

Figure 1 summarises the main cost drivers of the programme by activity type. Home visits—or household counselling for nutrition—were the greatest share of total costs (37%). This was followed by home visits for agriculture/poultry extension, site supervision, and training (12% each), monitoring and evaluation (9%) and community events (5%). Less than 5% of costs were allocated to planning, overhead, integration and coordination, material development, management, procurement and input distribution.

The main costs disaggregated by input category were dominated by personnel (68%), 48% of which came from hired labour. The remaining 20% of personnel costs represent beneficiary opportunity costs. This category was followed by travel/per diems (12%), supplies (7%), contracted services (6%) and overhead (6%).

**Figure 2:**
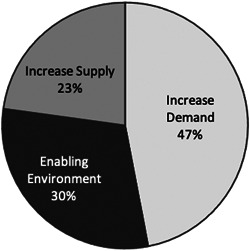
Cost drivers mapped to the nutrition‐sensitive value chain typology for the TRAIN intervention in Bangladesh (Source: Authors). TRAIN, Targeting and Realigning Agriculture to Improve Nutrition.

Programme activities were then mapped to a NSV chain typology (Figure 2). Results show that almost half of programme costs were incurred by demand‐side components. An example of a demand‐side component is nutrition BCC delivered through home counselling. Supply‐side intervention components accounted for 23% of overall costs. Supply‐side components include diversification and promotion of nutritious food production through agricultural extension. Thirty per cent of costs were associated with activities promoting the enabling environment. In this case, the enabling environment activities supported women's empowerment. Shared programme costs were allocated proportionally to the above three typologies. Proportional allocation was based on the percentage of frontline worker time assigned to agriculture, nutrition and gender components.

### 3.2 Costs by treatment arm

Total incremental costs per household per year ranged from a minimum of USD$36.62 (arm 2, only nutrition components) to a maximum of USD$87.50 for arm 4, which included all programme components (nutrition, nutrition‐sensitive agriculture, and gender). Arm 3 included two components—nutrition and nutrition‐sensitive agriculture, and fell in between at USD$65.18. As many activities occurred at the household level, the programme was designed to target more than one household member. Considering both the index  female and male in the household, the cost per household drops to USD$19.61, USD$34.25 and USD$47.13 by arm, respectively (Table 2).

**Table 2:** Summary of unit costs for the TRAIN intervention in Bangladesh (USD, 2019)

A. Number of beneficiaries				
Type of beneficiary	Total number of beneficiaries	Number of beneficiaries in Arm 2	Number of beneficiaries in Arm 3	Number of beneficiaries in Arm 4
Total number of households	3780	1260	1260	1260
Total number of index male/female HH members	7090	2353	2398	2339
Total number of index adults plus children	11,110	3703	3703	3703
Total household members (all)	17,653	5884	5884	5884

B. Unit costs				
	Overall	Arm 2	Arm 3	Arm 4
Total incremental cost ($USD)	$795,040.34	$153,817.18	$273,743.60	$367,479.57
Whole project period (40 months)				
Unit cost per household	$210.33	$122.08	$217.26	$291.65
Unit cost per index male and female	$112.14	$65.37	$114.15	$157.11
Unit cost per HH member (index male and female plus children)	$71.56	$41.54	$73.92	$99.23
Unit cost per household member	$45.04	$26.14	$46.52	$62.45
Per year (12 months)				
Unit cost per household	$63.10	$36.62	$65.18	$87.50
Unit cost per household index male and female	$33.64	$19.61	$34.25	$47.13
Unit cost per HH member (index male and female plus children)	$21.47	$12.46	$22.18	$29.77
Unit cost per household member	$13.51	$7.84	$13.96	$18.74

Abbreviation: HH, Household.

### 3.3 Sensitivity analyses

**Figure 3:**
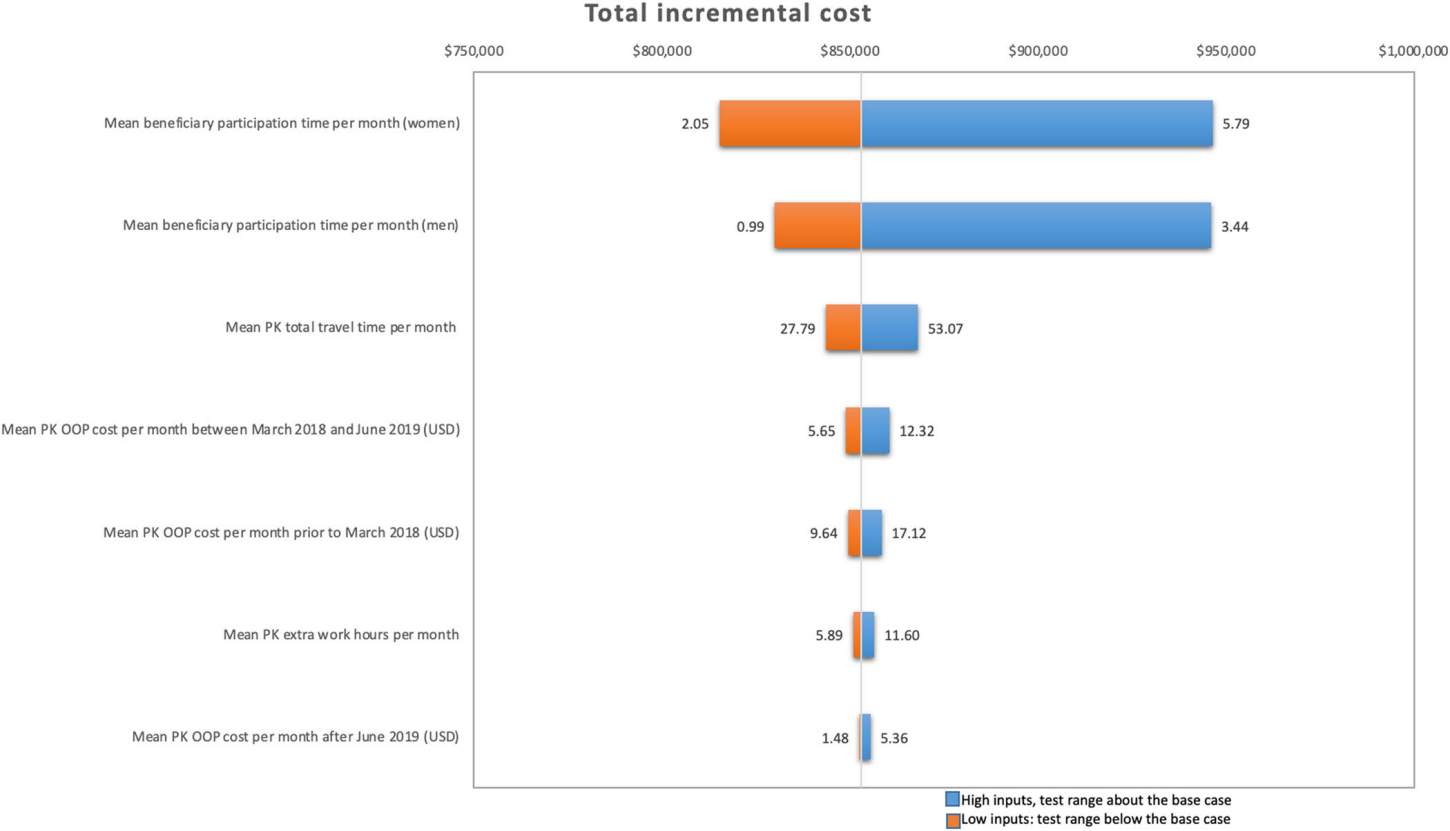
Tornado plot from the sensitivity analysis for total incremental costs

The tornado diagram shown in Figure 3 shows the impact that varying inputs has on the results. The diagram displays the most influential parameter on the top and moving down in order of importance. Mean beneficiary time for men and women has the largest impact on the variation in costs, followed by average total travel time per month. Varying these parameters results in a range of economic costs from $168,580 to $804,307, total incremental costs from $767,159 to $1,402,886 and average annual unit costs per household between $61 and $111 (Table 4). In all cases, the base case estimates were below the lower limit. Given the large variations in beneficiary participation time, costs could increase with time‐intensive activities, especially for women, who spend more time in programme activities. Variations in PK overtime and OOP costs do not affect total incremental costs and annual unit costs substantially (3.1% and 2.1%).

## 4 DISCUSSION

Integrated agriculture and nutrition interventions can provide effective platforms to reach vulnerable populations (Ruel et al., 2018). Despite progress in generating evidence on the effectiveness of these programmes, important gaps remain on intervention costs. This study offers evidence of the total incremental costs and costs per beneficiary of delivering a multisectoral agriculture‐nutrition programme through a microcredit platform in Bangladesh. Microfinance programmes have been lauded for expanding access to financial services to the poor but have also generated mixed results (Amin et al., 2003; Banerjee et al., 2015). That said, the reach of microfinance programmes—BRAC reaches 126 million people—presents a promising delivery platform for other services targeted to the vulnerable, such as agricultural extension and nutrition behaviour communication change. This study provides insights into the potential costs of such integrated programmes using a microfinance platform including granular details of disaggregated costs including the total incremental financial and economic costs by implementation arm and programme cost drivers by input and activity type.

For intervention unit costs, the average incremental cost per household was USD$63.10 regardless of the treatment arm. Similar to most nutrition‐sensitive programmes, TRAIN provided benefits beyond the targeted beneficiaries. Factoring in others in the household, unit costs are considerably lower. To gauge the potential range of unit costs, we calculated alternative scenarios for costs per beneficiary based on the average size of participating households. When considering the index female and male as the direct beneficiaries, the unit cost decreases to USD$33.64. When considering the index parents and all children^5^ in the household, the unit cost decreases to USD$21.47. Finally, when considering all household members as beneficiaries, the unit cost drops to USD$13.51. The results of the process evaluation suggest the intervention may benefit multiple household members (Siraj et al., 2019). Further evidence of this may be provided by the impact evaluation of the intervention, which is currently concluding endline data collection.

Unsurprisingly, total incremental costs and unit costs vary by the level of implementation intensity across the three intervention arms. With an increase in training intensity (frequency of sessions) and/or programme complexity (number of components), the unit cost per household increases. These increases are driven by personnel costs for additional activities such as agriculture extension or gender sessions (Arms 3 and 4). Similarly, beneficiary opportunity costs increased with programme complexity. Opportunity costs increased because beneficiaries spent more time in community events; home visits are longer with additional counselling components. Our results showed the index beneficiary women had higher opportunity costs across all treatment arms in all three scenarios; nearly double the costs of their husbands. This is likely because household visits targeted women. However, if programme impacts do not differ significantly by treatment arm, additional complexity may not be worth the cost.

We also examine the costs of incorporating women's empowerment activities. Women's empowerment activities accounted for 30% of programme costs, as part of facilitating the enabling environment for nutrition. As noted, the unit cost per household increased with programme complexity. Thus, Arm 4, which has the most activities, including gender forums and men's sensitisation, had the highest unit costs per beneficiary. Women spent almost twice the amount of time than men; our sensitivity analysis emphasises the range of higher opportunity costs for women (Table 3). This raises the concern of the time burden of programmes targeted at women. Recent evidence shows that nutrition‐sensitive programmes may impact women's time use (Margolies, 2019); but do not necessarily negatively impact nutrition (van den Bold et al., 2021). The impact evaluation will measure the effects on women's empowerment with the project‐level Women's Empowerment in Agriculture Index (pro‐WEAI)^6^ and on men's nutrition knowledge. The WEAI (Malapit et al., 2019) measures domains of intrinsic, instrumental and collective agency. The Pro‐WEAI measures women's time in work balance, revealing if opportunity costs are justified. Clearly, a significant proportion of costs were dedicated to activities to promote women's empowerment; endline data can show if they were cost‐effective, such as through a cost‐per‐unit increase in scores.

**Table 3:** Unit cost range estimates from the sensitivity analyses (USD, 2019)

Parameters to vary	Mean	SD	Assumed distribution
1. PK overtime			
Extra work hours per month (excluding travel)	4.24	2.34	Gamma
Total travel time per month	19.65	10.28	Gamma
2. PK out‐of‐pocket cost (subtracted travel stipend)			
OOP cost per month before March 2018 (USD)**—**no stipend	6.55	3.02	Gamma
OOP cost per month between March 2018 and June 2019 (USD)—with 200 BDT stipend	4.31	2.78	Gamma
OOP cost per month after June 2019 (USD)—with 500 BDT stipend	1.45	2	Gamma
3. Beneficiary participation time per month in hours			
Men	0.96	1.22	Gamma
Women	1.8	1.66	Gamma

Abbreviations: BDT, Bangladesh Taka; HH, household; OOP, out‐of‐pocket costs; PK, Pushti Kormi (nutrition frontline worker); SD, standard deviation.

**Table 4:** Sensitivity analysis results of varying key inputs on total economic and unit costs

Simulation results (5000 simulations)			
Outcome Variables	Base case	Lower limit	Upper limit
Total economic cost	$148,115.30	$168,579.72	$804,307.08
Total incremental cost	$746,694.21	$767,158.62	$1,402,885.99
Annual unit cost by household	$59.26	$60.89	$111.34
Annual unit cost by index male and female	$31.59	$32.46	$59.36
Annual unit cost by household member (index male/female plus children)	$20.16	$20.72	$37.88
Annual unit cost per household member	$12.69	$13.04	$23.84

Examining costs by activity, the main driver was household nutrition counselling. Coupled with the agricultural household visits, training, and community events, beneficiary outreach accounted for over half of the costs. This finding reflects the intensive nature of activities to reach beneficiaries. Examining costs by input, the main cost driver was personnel. Personnel costs were driven by frontline workers, accounting for over two‐thirds of total personnel costs. Salary data were gathered from BRAC expenditures. We also estimated frontline workers economic costs in overtime work and OOP costs. Our analysis found variations in opportunity costs that have important implications for scale‐up and sustainability. Among frontline workers, PKs spent a substantial amount of their time and resources conducting their work. These costs could limit the ability of PKs to conduct their activities in the long run. BRAC addressed this constraint by increasing PK travel stipends over time.

We then mapped costs to the NSV chain typology. We found similar results to the first programme costed with the SEEMS approach: a nutrition‐sensitive agriculture programme, with agriculture and nutrition BCC delivered through community‐based preschools in Malawi (Margolies et al., 2021). In both TRAIN and the Malawi programme, over 50% of costs came from the demand‐side pathway, approximately a quarter of costs from supply‐side activities and the remaining amount from supporting the enabling environment. Though these are just two studies, they may suggest that integrated agriculture interventions with an explicit focus on improving maternal and child nutrition through BCC are more likely to direct resources to increase the demand for nutritious foods rather than the supply. Building on these findings is an important priority for ongoing research. Further, the use of the NSV chain typology alongside the SEEMS standardised approach facilitates comparisons between different types of nutrition‐sensitive interventions. This aids funders, policymakers and implementers to better understand relative costs and associated potential tradeoffs in intervention design, and supports donor negotiations for resource mobilisation across nutrition, health, and agriculture sectors.

Currently, comparisons with the literature on the costs of integrated agriculture and nutrition interventions are limited. These limitations are due to the small number of studies available, the heterogeneity in intervention design and the absence of a standardised methodology capturing activities across sectors. We found that for TRAIN, the incremental cost per household is within the range in the literature. The costs of an intervention promoting orange‐fleshed sweet potato (OFSP) production and consumption in Kenya were USD$155 per pregnant woman or USD$110 per beneficiary when mothers and infant children were included (C. E. Levin et al., 2019). Another programme promoting OFSP cost $146 per household in Mozambique and $132 in Uganda; $65 per individual beneficiary in Mozambique and $49 in Uganda (de Brauw et al., 2018). A study in Cambodia estimated the costs over ten years of a homestead production intervention at USD$929 per household (Dragojlovic et al., 2020). In Zimbabwe, the costs per household for a programme providing community gardens for people with HIV were USD$1890 (Puett et al., 2014). A cross‐country study in Ethiopia, Nigeria and India modelled costs per child reached for 12 agriculture‐nutrition interventions. Modelled costs ranged widely from USD$0.58 for a media and education campaign to USD$2650 for a livestock programme (Masters et al. 2018). While these studies assess costs for different types of interventions and outputs (i.e., individual beneficiaries or households reached), the unit costs generated are critical for decision‐makers to assess the affordability of multisectoral nutrition for a given intervention and country context. They also demonstrate the need for improved guidance to generate standardised cost estimates to increase comparability and generalisability.

Incorporating frontline worker opportunity costs in the TRAIN cost analysis highlights the programme's sustainability concerns. Despite the travel stipend provided in the second year, frontline workers shouldered additional OOP costs given the intensive nature of household‐level interventions. SEEMS‐Nutrition is underlining such questions in building evidence on the costs and cost‐effectiveness of nutrition‐sensitive interventions. This study presents full programme costs in the pre‐COVID period and is an important step toward a comprehensive economic evaluation. As noted above, a recent systematic review has found that nutrition‐sensitive agricultural interventions have a significant positive impact on dietary diversity among children 6−60 months old (Margolies et al. 2022). This paper helps illustrate the costs of these complex interventions, providing a benchmark that can be used to assess costs and affordability of this and similar programmes. Importantly, the methods outlined here provide a template for future cost analyses and the results provide the groundwork for meaningful comparisons among multisectoral programmes. Lastly, the economic costs, which include both the financial and economic costs of all implementing partners, government and participants, can provide insights into the sustainability of the programme, and will be combined with a forthcoming study on the programme's impact on nutrition outcomes to generate evidence on cost‐effectiveness.

## 5 CONCLUSIONS

This study presents the financial and economic incremental costs of implementing an integrated agriculture‐nutrition intervention through a micro‐credit platform in Bangladesh. Cost‐per beneficiary estimates compare favourably with multisectoral nutrition‐sensitive interventions implemented through different platforms. These results demonstrate that a standardised approach for measuring the costs of multisectoral nutrition strategies enhances comparability and transparency, increasing the application of cost data for assessing affordability for use in evaluation, planning and policymaking.

## AUTHOR CONTRIBUTIONS

Giang Thai led data collection and cost analyses and contributed to the manuscript. Amy Margolies contributed to the cost study methodology, conducted cost analyses and led drafting of the manuscript. Aulo Gelli contributed to the cost study methodology and to conception and design, provided technical advice, drafted and reviewed manuscripts. Nasrin Sultana co‐led data collection and reviewed the manuscript. Esther Choo contributed to the data analysis and reviewed the manuscript. Neha Kumar reviewed the manuscript. Carol Levin contributed to the cost study methodology and to conception and design, provided technical advice, participated in data collection, edited the manuscript and reviewed manuscripts.

## CONFLICT OF INTEREST

The authors declare no conflict of interest.

## Supporting information

Supplementary information.

## Data Availability

Data available on request from the authors.

## Footnotes

1 Microcredit is a financial service offered by microfinance programmes, which target the poor and others unable to access traditional banks.

2 BRAC. http://www.brac.net/sites/default/files/microfinance.pdf.

3 The index husband is the spouse of the primary female respondent.

4 Source: https://www.xe.com/currencyconverter/convert/?Amount=1%26From=USD%26To=BDT, November 2019.

5 Index parents and children include all children listed from the RCT survey roster, which could potentially include children of the primary respondent female from a prior marriage, for example.

6
https://weai.ifpri.info/versions/pro-weai/
