# Supplementary material for: The economic costs of a multisectoral nutrition programme implemented through a credit platform in Bangladesh
Source: Matern Child Nutr. 2022 Oct 18;19(1):e13441. doi: 10.1111/mcn.13441 (PMC9749601; doi:10.1111/mcn.13441)
Supplement: Supplementary file 1 — Supplementary information. [file MCN-19-e13441-s001.docx]

# Appendix

Appendix Figure 1. TRAIN intervention design (Building Resources Across Communities (BRAC), 2017)


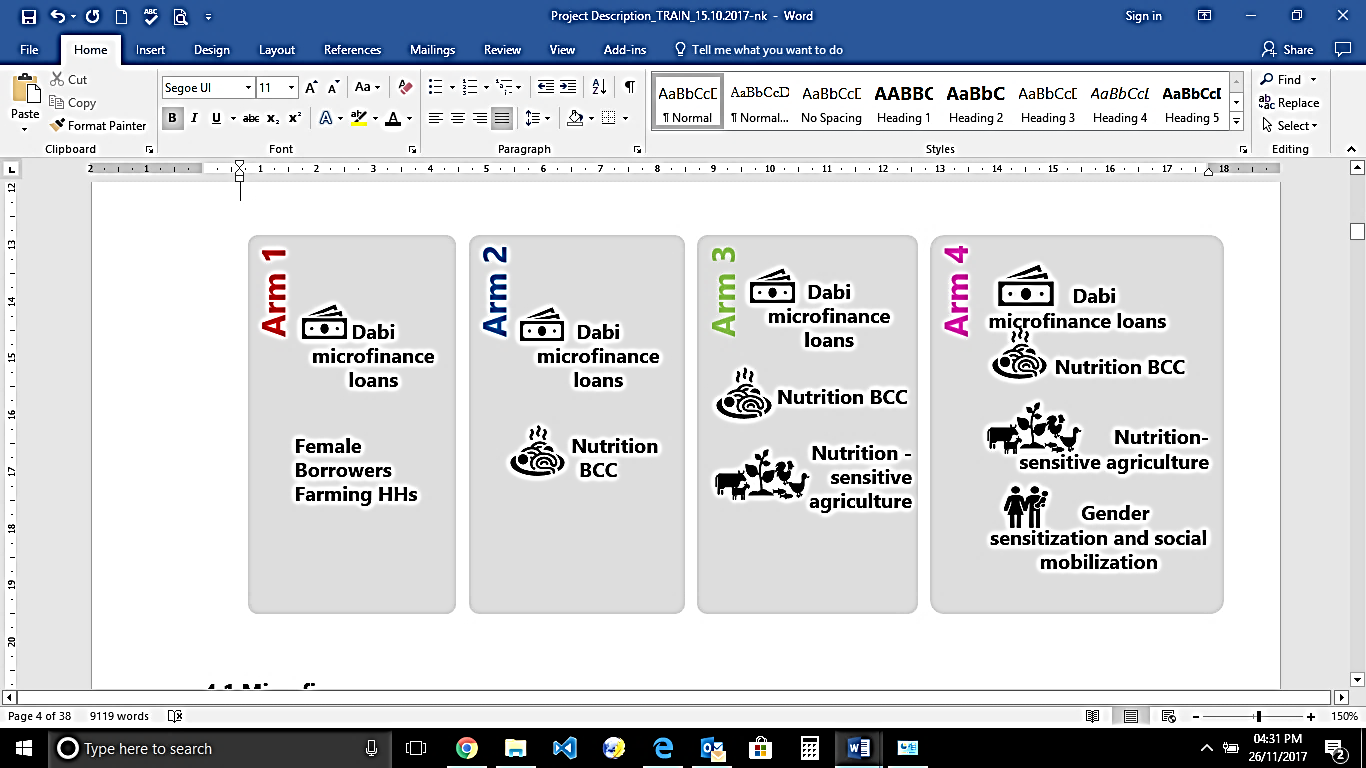


Appendix Table 1: SEEMS unit cost framework for TRAIN (Source: Authors)

Appendix Table 2. TRAIN Activities Mapped to SEEMS Standardized Activity Categories

| **Activity** | **Definition** |
| --- | --- |
| **START UP COSTS** |  |
| Program installation | Frontline worker recruitment, household selection |
| Awareness raising/sensitization | Meeting with local government officials at the beginning of the project |
| Training | Gender sensitization, nutrition BCC and agricultural trainings for frontline workers |
| Materials development | Development of nutrition, agriculture and gender messaging materials |
| **RECURRENT COSTS** |  |
| Planning/microplanning | Monthly meetings with frontline workers at the end of each month to make plan for the following month |
| Management | BRAC head office staff meetings with frontline workers to keep track of project implementation |
| Monitoring and Evaluation | Monthly checks of frontline worker diaries and reports |
| Procurement | Procurement of inputs for frontline workers and households |
| Distribution of inputs | Distribution of inputs to frontline workers |
| Site supervision | BRAC head office staff field visits to check on project implementation |
| Home visits: household counseling | Monthly nutrition counseling, gender counseling |
| Home visits: agriculture/poultry extension | Technical support for homestead nutri-gardens |
| Community events/extension | Agricultural extension sessions, gender sensitization couples sessions |
| Establishing and running community groups | Household mobilization to attend community events |
| Integration and coordination | Meetings for BRAC staff across agriculture, gender and nutrition areas |
| Indirect/overhead | Office maintenance, office rent |

Appendix Table 3. TRAIN Input Categories Mapped to SEEMS Standardized Input Categories

| **SEEMS Input category** | **TRAIN Input Description** |
| --- | --- |
| **Personnel** | BRAC staff paid time (Head Office, Sub-District, Field Staff, Frontline workers) |
| **Supplies** | Office supplies and other non-agriculture supplies |
| **Agriculture supplies** | Vegetable seeds |
| **Agricultural equipment** | Planting equipment |
|  |  |
| **Contracted services** | Training-related venue, training venue utilities |
| **Travel/per diem/allowances** | Travel allowances, per diems |
| **Overhead/indirect** | Office and equipment maintenance, rent, utilities, communication, etc. |

Appendix Table 4. Nutrition-sensitive Value (NSV) Chain Activity Coding

| NSV Intervention Typology | Entry Point | Activity |
| --- | --- | --- |
| Increase supply | Diversification/promotion of nutritious crops | - Materials development (extension training) - Home visits: agricultural/poultry extension - Provision of agricultural inputs - Community events: agricultural extension - Agricultural training for frontline workers |
| Increase demand | Behavior Change Communication | - Home visits: nutrition counseling - Materials development (nutrition behavior communication change training) - Provision of nutrition inputs (measuring plates, etc.) - Nutrition training for frontline workers |
| Enabling Environment | Women’s Empowerment | - Home visits: gender - Materials development (gender) - Establishing and running community groups (gender) - Community events: gender - Procurement and distribution of inputs (gender materials) - Gender training for frontline workers |
| Shared program costs allocated to above three typologies | Coordination/Support | - Program installation - Site supervision - Indirect/overhead - Planning/microplanning - Integration and coordination - Monitoring and evaluation - Awareness raising/sensitization - Management |

Appendix Table 5. Summary of total incremental costs by treatment arm for the TRAIN intervention (USD 2019)

|  | **Arm 2** | **Arm 3** | **Arm 4** | **Arm 2** | **Arm 3** | **Arm 4** |
| --- | --- | --- | --- | --- | --- | --- |
| **Input** |  |  |  |  |  |  |
| Personnel (hired) | $77,301.65 | $131,657.00 | $ 172,710.14 | 50% | 48% | 47% |
| Personnel (beneficiaries) | $25,192.04 | $55,438.55 | $81,705.16 | 16% | 20% | 22% |
| Supplies | $11,217.51 | $19,335.83 | $25,696.90 | 7% | 7% | 7% |
| Agriculture supplies | $437.98 | $754.96 | $1,003.32 | 0% | 0% | 0% |
| Agriculture equipment | $1.16 | $2.00 | $2.65 | 0% | 0% | 0% |
| Contracted services | $9,801.64 | $16,895.27 | $22,453.45 | 6% | 6% | 6% |
| Fuel and maintenance | $3.93 | $6.77 | $9.00 | 0% | 0% | 0% |
| Travel/per diem/allowances | $20,615.19 | $33,715.57 | $42,718.15 | 13% | 12% | 12% |
| Overhead | $9,246.08 | $15,937.64 | $21,180.78 | 6% | 6% | 6% |
| **Total:** | **$153,817.18** | **$ 273,743.60** | **$ 367,479.57** | **100%** | **100%** | **100%** |
| **Stage** |  |  |  |  |  |  |
| Start-up | $7,568.05 | $13,045.19 | $17,336.78 | 5% | 5% | 5% |
| Recurrent | $146,249.13 | $260,698.41 | $350,142.79 | 95% | 95% | 95% |
| **Total** | **$153,817.18** | **$273,743.60** | **$367,479.57** | **100%** | **100%** | **100%** |
| **Activity** |  |  |  |  |  |  |
| Planning/microplanning | $6,798.56 | $11,514.58 | $15,122.50 | 4% | 4% | 4% |
| Program Installation | $233.57 | $402.61 | $535.05 | 0% | 0% | 0% |
| Awareness raising/sensitization | $217.35 | $374.66 | $497.91 | 0% | 0% | 0% |
| Training | $15,571.58 | $33,688.25 | $43,015.25 | 10% | 12% | 12% |
| Materials development | $2,160.47 | $3,724.05 | $4,949.18 | 1% | 1% | 1% |
| Management | $896.07 | $1,544.56 | $2,052.69 | 1% | 1% | 1% |
| Monitoring and evaluation | $15,457.93 | $25,930.34 | $33,830.43 | 10% | 9% | 9% |
| Procurement | $1,341.06 | $2,311.61 | $3,072.08 | 1% | 1% | 1% |
| Distribution of inputs | $1,007.11 | $1,735.98 | $2,307.08 | 1% | 1% | 1% |
| Site supervision | $18,566.48 | $32,003.39 | $42,531.81 | 12% | 12% | 12% |
| Home visits: nutrition/gender | $64,700.62 | $98,052.15 | $127,943.45 | 42% | 36% | 35% |
| Home visits: agriculture/poultry extension | $12,029.82 | $36,972.41 | $44,370.88 | 8% | 14% | 12% |
| Community events/extension | $6,010.55 | $10,360.51 | $27,216.57 | 4% | 4% | 7% |
| Integration and coordination | $2,327.90 | $3,927.60 | $5,148.90 | 2% | 1% | 1% |
| Indirect/overhead | $6,498.11 | $11,200.91 | $14,885.77 | 4% | 4% | 4% |
| **Total:** | **$153,817.18** | **$273,743.60** | **$367,479.57** | **100%** | **100%** | **100%** |

Appendix Table 6. Sensitivity analysis using gamma distribution for inputs

| **Variable tested through sensitivity analysis** | **Shape parameter** | **Scale parameter** |
| --- | --- | --- |
| Mean extra work hours per month | 3.28 | 1.29 |
| Mean OOP cost per month prior to Mar18 (USD) | 4.70 | 1.39 |
| Mean OOP cost per month between Mar18 and Jun19 (USD) | 2.40 | 1.79 |
| Mean OOP cost per month after Jun19 (USD) | 0.53 | 2.76 |
| Mean total travel time per month | 3.65 | 5.38 |
| Mean beneficiary participation time per month (men) | 0.62 | 1.55 |
| Mean beneficiary participation time per month (women) | 1.18 | 1.53 |

Appendix Table 7. Qualitative costing interview methods

| Interview type | Interviewee | Length (min) | Number interviews | Participants per interview | Total Participants | Selection criteria |
| --- | --- | --- | --- | --- | --- | --- |
| Data collection Round 1 | | | | | | |
| Key informant interview | BRAC Head Office Staff | 60 | 3 | 1 | 3 | Involved in program implementation |
| Key informant interview | Frontline workers (PK) | 120 | 3 | 1 | 3 | One per treatment arm |
| Focus Group Discussion | Field Organizers (FO) | 150 | 1 | 6 | 6 | Districts with all 3 treatment arms* |
| Focus Group Discussion | District Managers | 150 | 1 | 5 | 5 | Districts with all 3 treatment arms |
| Data collection Round 2 | | | | | | |
| Key informant interview | BRAC Head Office Staff | 60 | 4 | 1 | 4 | Involved in implementation & financial mgt. |
| Focus Group Discussion | Field Organizers (FO) | 120 | 1 | 2 | 2 | Districts with all 3 treatment arms |
| Focus Group Discussion | District Managers | 120 | 1 | 5 | 5 | Districts with all 3 treatment arms |
| Focus Group Discussion | Frontline workers (PK) | 150 | 7 | 3 FGDs Rangpur (n=4)  2 FGDs Dhaka (n=2)  2 FGDs Khulna (n=4) | 24 | 3-4 per treatment arm in each division |

*One FO was randomly selected for interview from each of the selected districts.

## Supplementary Information on Data Collection

## Estimating personnel costs for BRAC Headquarters staff, District Managers (DM) and Field Officers (FO)

The gross salaries plus benefits for BRAC headquarters staff, District Managers and FOs are equivalent to their economic costs, as these staff are fully reimbursed for all out-of-pocket costs incurred while conducting program activities. BRAC financial expenditure data reported salaries for BRAC national staff, DMs and FOs. District Managers and BRAC headquarters staff divide their time across various projects. Thus, DMs, FOs and HQ staff financial costs are the proportions of their weekly time allocated for the TRAIN project. These proportions were determined through key informant interviews and focus group discussions with head office staff, DMs and FO’s (Appendix Table 7).

## Frontline worker labor costs

Pushti Kormi (PK) working hours are six hours per day, six days per week. Interviews with PKs in arm 3 (Nutrition BCC + Agriculture Extension) and PKs in arm 4 (Nutrition BCC + Agricultural Extension + Gender Sensitization), indicated that frontline workers spent more time on program activities than the salaried 36-hour week. PKs incurred additional costs due to the number of activities required during household visits, especially for arms 3 and 4, along with additional travel time and out-of-pocket expenses to reach TRAIN households. In the first year of the program, PK economic costs varied by treatment arm and as a result of seasonality (weather affected travel), geographical distance and regional topography. The socioeconomic status of individual frontline workers also influenced transportation choices due to the cost of different methods of transport.

Our calculation of total OOP expenses for all PKs is extrapolated from the subsample of PKs we collected in both rounds of data collection. Focus group interviews (n=7) and key informant interviews (n=3) with PKs were used to estimate the economic costs for all frontline workers (total PKs n=108) (Appendix Table 7). We assumed that only 50% of PKs put in extra hours, recognizing that not all PKs in arms 3 and 4 may have logged extra work hours. These estimates reflect average frontline workers’ time and expenses incurred up until March 1^st^, 2018. In the first year of the program, PKs did not receive stipends for transport, food or mobile phone costs. However, in the second year of implementation (March 2018), BRAC provided a 200 BDT monthly stipend to PKs to cover the extra transport, food and communication costs of TRAIN activities (i.e., additional time spent during household visits, providing logistical support for FOs during community events). BRAC then raised the stipend to 500 BDT in June 2019. The rates set by BRAC for stipends assumed that all PK OOP and travel costs would be covered, regardless of distance traveled to households.

## Beneficiary economic costs

Beneficiary costs are estimated using time allocation data from the process evaluation of the TRAIN intervention. The process evaluation targeted a subsample of female participants and their husbands who were asked about their time spent on TRAIN activities specific to each treatment arm as well as on any costs incurred for their participation in the program. Since the survey questions specifically targeted PK home visits in the last 30 days and FO training in the last 12 months, we made the assumption that there was little variation in beneficiary participation throughout the year for PK visits and throughout the 2-year period for FO-led trainings. Thus, for each activity, average time recorded last month (or last year) was scaled up to the full project duration. The economic costs of the beneficiaries' time for each activity are calculated using agricultural wage data from the process evaluation. The average costs per activity and out-of-pocket expenses per female and male beneficiaries in each treatment arm are then extrapolated to all beneficiaries in the same treatment arm. We assume is that there is limited variation in beneficiaries’ participation time. Total out-of-pocket costs are added to the total financial costs. The total economic costs of beneficiary time, aggregated from each activity, are part of the total economic costs.

The entire household was targeted for TRAIN program activities. However, household members directly involved in TRAIN activities were the index female respondent and her spouse (index husband). Thus, the beneficiary opportunity costs only take into account the participation of the index woman and her husband. We were not able to gather information on resource use from other household members, such as mother-in-laws or adolescents who may have provided childcare during home visits or community events.
